# GaN intermediate band solar cells with Mn-doped absorption layer

**DOI:** 10.1038/s41598-018-27005-z

**Published:** 2018-06-05

**Authors:** Ming-Lun Lee, Feng-Wen Huang, Po-Cheng Chen, Jinn-Kong Sheu

**Affiliations:** 1 Department of Electro-Optical Engineering, Southern Taiwan University of Science and Technology, Tainan City, 71005 Taiwan; 2 Department of Photonics and Advanced Optoelectronic Technology Center, National Cheng Kung University, Tainan City, 70101 Taiwan

## Abstract

The effect of Mn concentration on the optical properties of Mn-doped layers grown by metalorganic vapor phase epitaxy is investigated. The Mn-doped GaN layers exhibite a typical transmittance spectrum with a distinct dip around 820 nm which is attributed to the transition of electrons between the edge of valence band and the Mn-related states within the bandgap. In addition, electroluminescence (EL) spectra obtained from the bipolar devices with Mn-doped GaN active layer also show that considerable Mn-related energy states existed in the bandgap. The position of the Mn-related energy states in the GaN is first evaluated via EL spectra. In addition to the absorption of band edge, the Mn-related energy states behaving like an intermediate band cause an additional sub-band gap absorption. Consequently, the fabricated GaN-based solar cells using Mn-doed GaN as the absorption layer exhibit photocurrent higher than the control devices without Mn doping. Under one-sun air mass 1.5 G testing condition, the short-circuit current of the Mn-doed GaN solar cells can be enhanced by a magnitude of 10 times compared with the cells without Mn doping.

## Introduction

The GaN-based materials doped with Mn have recently attracted considerable attention because they exhibit ferromagnetism above room temperature (RT)^1–3^. This property can potentially provide new functionalities and enhanced performance in conventional electronic devices operating at RT^4–7^. However, RT-operated spintronic devices made of Mn-doped GaN materials is still rare. The experimental results revealed that Mn-doped GaN exhibits a paramagnetic property when the Mn atomic concentration exceeds a certain value^8^. Theoretical work conducted by Martí *et al*. has predicted that In_x_Ga_1−x_N-based solar cell doped with Mn as an intermediate band (IB) material could achieve a maxima efficiency of 53.4% when the Mn atomic concentration exceeds 6 × 10^19^ cm^−3 ^^9^.Therefore, interest in the Mn-doped GaN has been driven by the theoretical proposal of an innovative type of solar cell, namely, an intermediate-band solar cell (IBSC)^9^. The IB concept has been proposed to improve the efficiency of solar cells^10–14^. Besides Mn-doped GaN materials, some experimental results on the properties of IB materials and solar cells, such as InAs/GaAs quantum dots^15–17^, diluted II-VI oxide semiconductors^18–20^, have been reported in the literature. However, an IBSC composed of GaN-based materials reported in the literature is still rare^20–22^. Many investigations conducted on Mn-doped GaN have shown that a Mn impurity band could form nearly in the middle of the GaN bandgap as the concentration of Mn in GaN exceeding a specific number^23–25^. The Mn-doping control in GaN is a crucial technology for spintronics and photovoltaic applications. The interface and junction properties strongly affect the performance of these devices with the Mn-doped layers. Thus, the control of specific Mn-doping profile of these junction devices is important. The Mn-doping control in GaN that depends on growth parameters is still lacking, although the significant diffusion and memory effect of Mn dopants in GaN grown by metalorganic vapor phase epitaxy (MOVPE) have been reported in the literature^26^. In this study, the effect of Mn doping concentration on the optical properties of Mn-doped GaN layers is systematically investigated, and GaN-based solar cells with Mn-doped absorption layer are also characterized.

## Methods

In this study, all the epitaxial samples were grown by a vertical metalorganic vapor phase epitaxy (MOVPE) system (EMCORE D-180). The epitaxial growth procedure started from the preparation of GaN template layers. The layer structure of GaN template consisting of a 30 nm-thick GaN nucleation layer grown at 560 °C and a 3 μm-thick unintentionally doped GaN (u-GaN) layer were grown at 1060 °C on c-face sapphire substrates. These wafers are denoted as u-GaN templates in the following paragraphs. The background electron concentration of the u-GaN templates was approximately 5 × 10^16^ cm^3^. In this study, all the Mn-doped GaN epitaxial layers used bismethylcyclopentadienyl manganese (CH_3_C_4_H_5_)_2_Mn as the Mn-doping source were grown on the u-GaN templates. Growth parameters, including growth temperature, pressure, and flow rate of the (CH_3_C_4_H_5_)_2_Mn, were varied to examine the effect of the epitaxial parameters on crystalline quality and optical properties of the Mn-doped GaN epitaxial layers. The details were described elsewhere^26^. Photoluminescence (PL) and transmittance were performed on the Mn-doped GaN samples to evaluate their optical properties. For PL measurements, the light source was He-Cd laser emitting at 325 nm. The PL signal was dispersed by a monochromator (Acton SP-500) and was detected by a thermoelectrically cooled photomultiplier tube (Hamamatsu R94302) using the photon-counting method. Secondary ion mass spectroscopy (SIMS) was performed to determine the doping levels of Mn atoms in the Mn-doped GaN epitaxial layers grown with different flow rates of (CH_3_C_4_H_5_)_2_Mn.

## Results and Discussion

**Figure 1:**
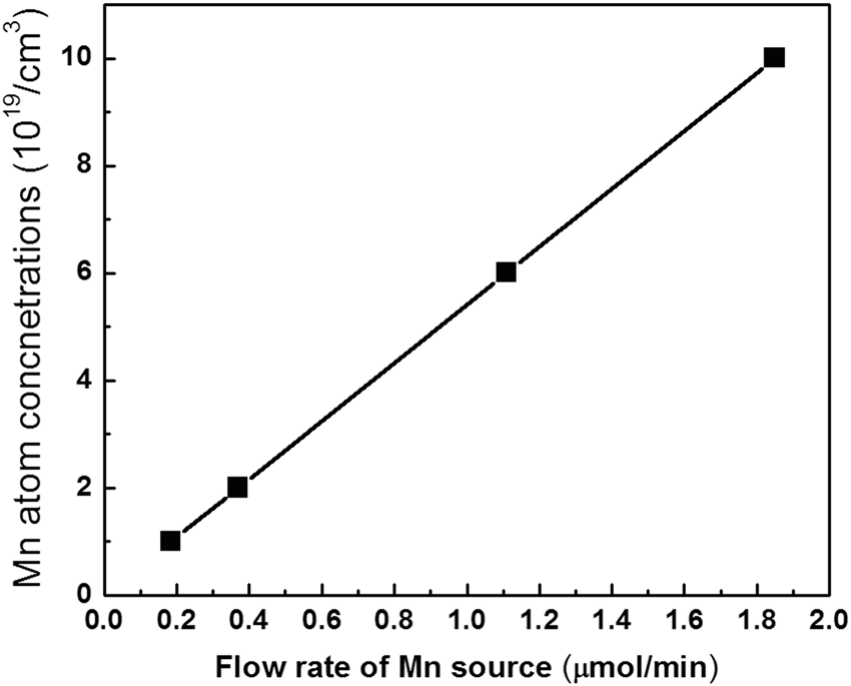
Mn ion counts of the Mn-doped GaN determined by SIMS as a function of the (CH_3_C_4_H_5_)_2_Mn molar flow rate.

Figure 1 shows the Mn doping concentration of Mn-doped GaN epitaxial layers determined by SIMS as a function of the (CH_3_C_4_H_5_)_2_Mn molar flow rates of 0.0185, 0.37, 1.11, and 1.85 µmol/min which the corresponding concentrations of Mn atom were 1 × 10^19^ and 2 × 10^19^, 6 × 10^19^, 1 × 10^20^ cm^−3^, respectively. In these wafers, other epitaxial parameters were fixed, except for the flow rate of Mn source. The growth temperature and pressure were 1000 °C and 100 torr, respectively. The Mn atoms incorporated into the GaN host crystal increased linearly with the (CH_3_C_4_H_5_)_2_Mn molar flow rate. In principle, the maximum dopant concentration that can be achieved in a semiconductor under a thermodynamic equilibrium is dominated by the solubility limit of dopant^27^. The data illustrated in Fig. 1 implies that the ambient impurity concentration (Mn) is lower than the solubility limit of Mn in GaN even when the flow rate is as high as 1.85 µmol/min. In other words, incorporation of Mn atoms could be well controlled by varying the source flow rate when it is not higher than 1.85 µmol/min.

Results regarding to the effects of the incorporation efficiency of Mn dopant in the GaN epitaxial layer grown by MOVPE were systematically investigated (Figures S1–S5, Supplementary Information). The experimental results showed that the Mn-doped GaN epitaxial layers grown with a relatively low growth pressure and temperature have a high incorporation efficiency of Mn atoms in the GaN layers. (Figures S1 and S2, Supplementary Information)

**Figure 2:**
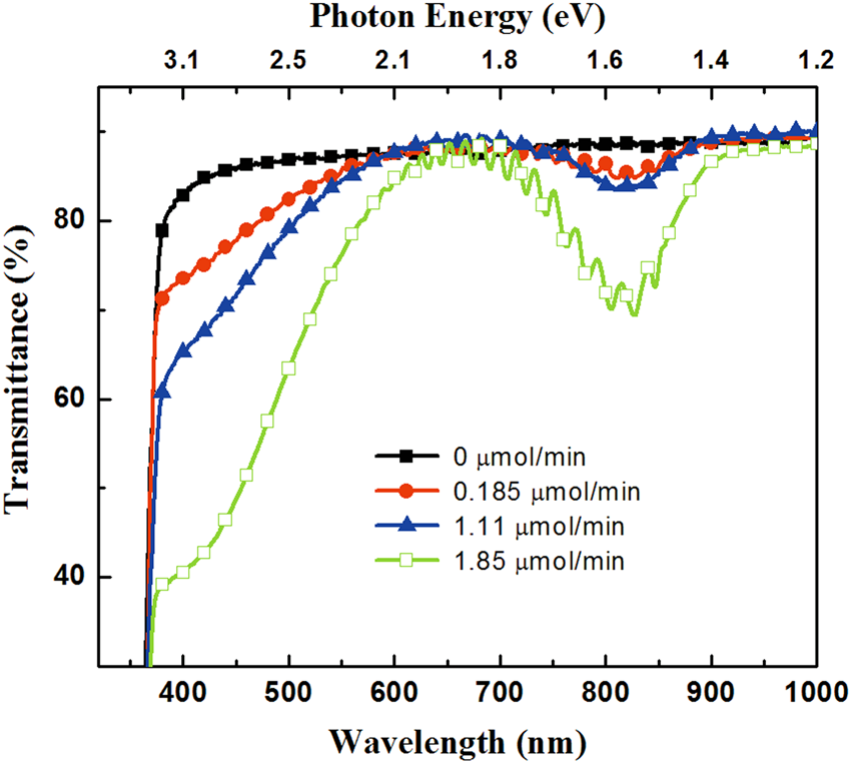
Typical transmission spectra obtained from the GaN epitaxial layers doped with the (CH_3_C_4_H_5_)Mn molar flow rates of 0, 0.185, 1.11, and 1.85 µmol/min.

**Figure 3:**
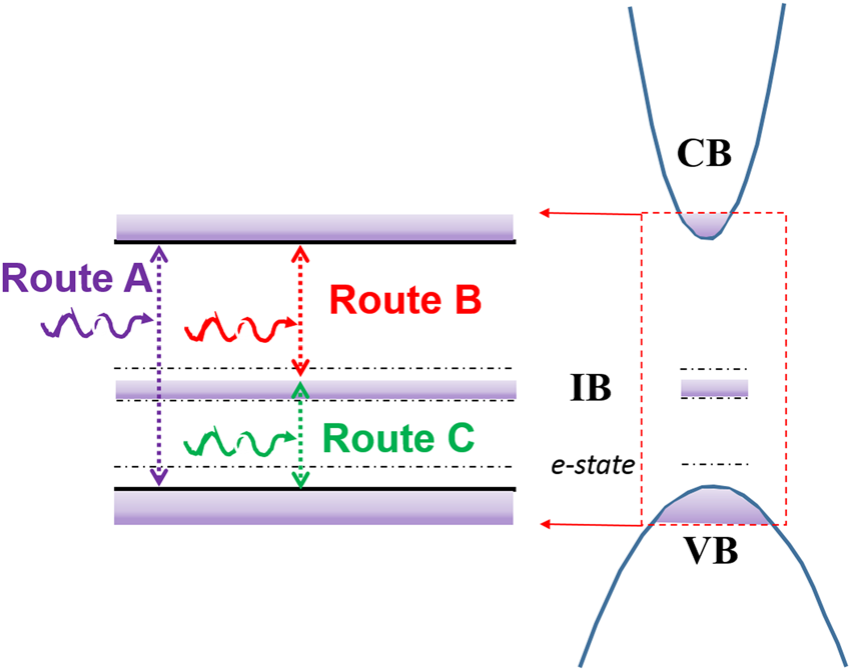
Schematic of the band of the Mn-doped GaN and possible absorption routes involving Mn IB.

**Figure 4:**
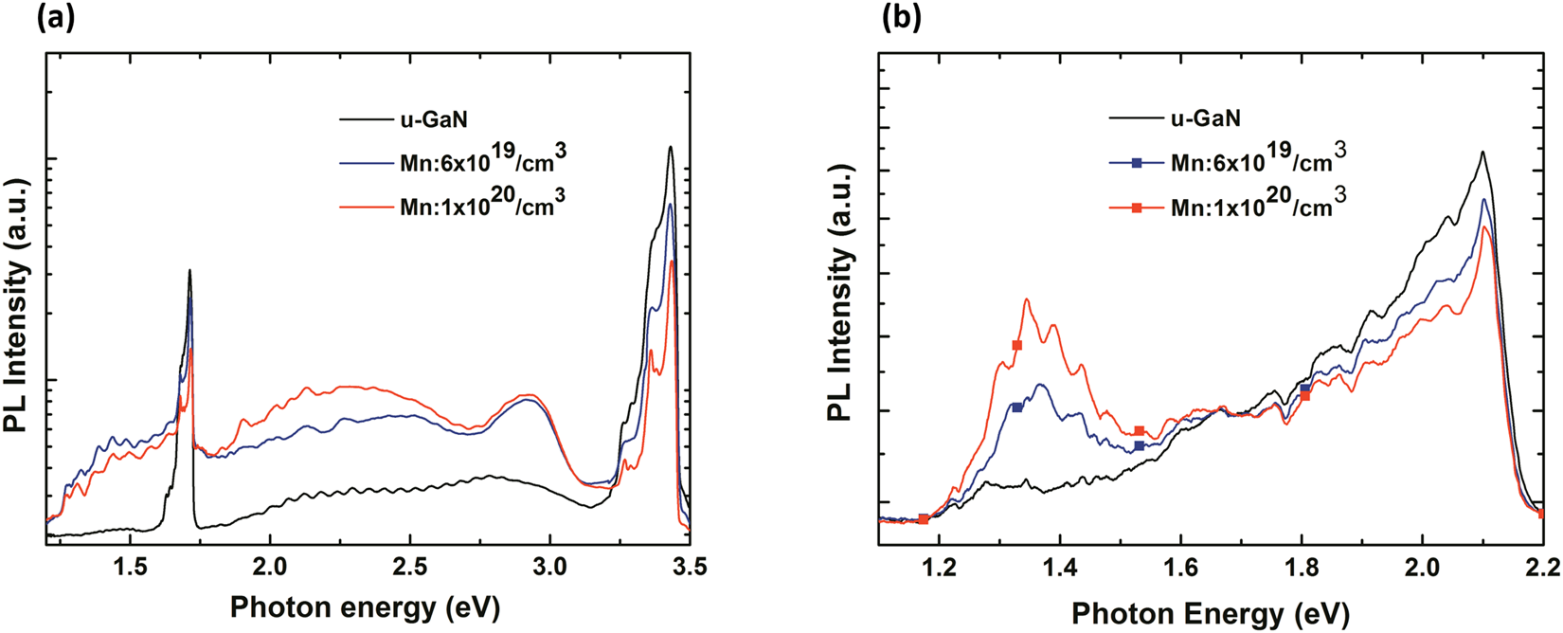
Typical PL spectra of the (**a**) u-GaN and (**b**) Mn-doped GaN with different Mn concentrations. The spectra were obtained at a temperature of 300 K.

**Figure 5:**
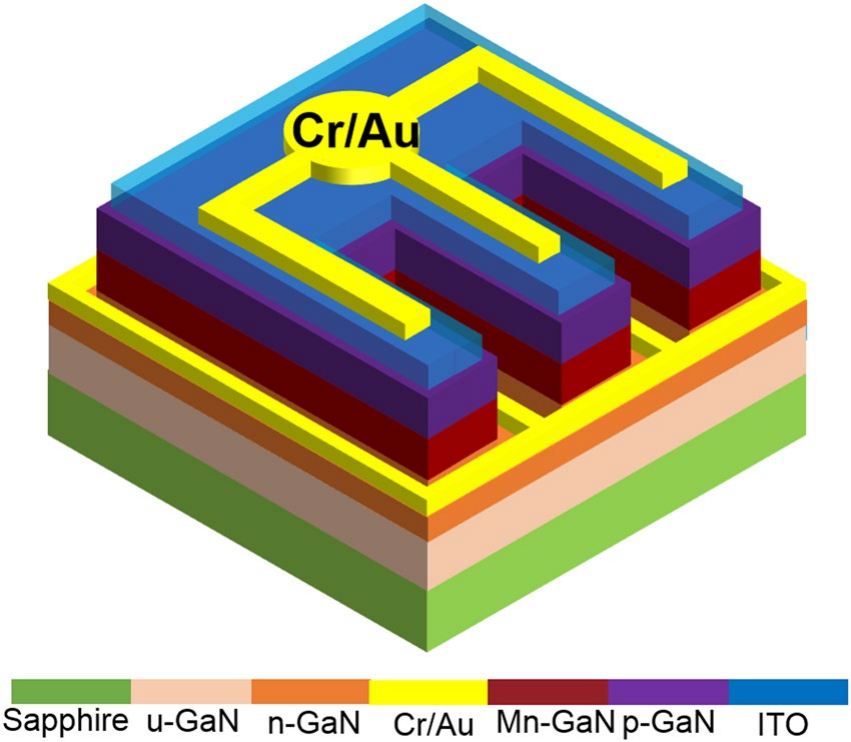
Schematic of the bipolar devices with the Mn-doped active layer.

Figure 2 illustrates the typical transmission spectra obtained from u-GaN and Mn-doped GaN samples with the (CH_3_C_4_H_5_)_2_Mn molar flow rates of 0, 0.185, 1.11, and 1.85 µmol/min. The transmission spectrum obtained from u-GaN films showed a steep transition edge at approximately 365 nm (i.e., the bandgap of GaN). However, the Mn-doped GaN films showed a strikingly different optical transmission spectrum compared with the undoped GaN films. A schematic of the band of the Mn-doped GaN and possible absorption routes (labeled A, B, and C) are depicted in Fig. 3 to elucidate the possible optical transitions in the Mn-doped GaN. The absorption edge at approximately 365 nm is denoted as absorption A, which is due to the transition from the valence band (VB) to the conduction band (CB) corresponding to an energy of 3.4 eV. The broad transition with a threshold of approximately 620 nm corresponding to an energy of 2.0 eV and a tail extending to 365 nm was denoted as absorption B. The presence of absorption B could be attributed to the electrons undergoing excitation from the Mn-related states within the bandgap to the CB. Meanwhile, an infrared absorption band centered at approximately 820 nm, denoted as absorption C, could be attributed to the transitions between the VB and the Mn-related states. Absorption B between the 365 and 650 nm observed via transmission spectroscopy from the Mn-doped GaN films is due to the transition between the Mn-related states and the CB. In addition, the broad infrared absorption band centered at approximately 820 nm ranging from 720 nm to 900 nm implies that the Mn-related energy states with a distribution width of 0.35 ± 0.05 eV, depending on the Mn doping concentration in the GaN layer. In this study, the u-GaN epitaxial layers grown on sapphire substrate exhibited an n-type conduction, and the typical electron concentration determined by Hall-effect measurement was approximately 5 × 10^16^ cm^−3^. The Hall effect measurements indicated that the sheet resistance of the Mn-doped GaN layers was extremely high exceeding 10^11^ Ω/square, which is the measurement limit of our setup, even when the doping flow rates of (CH_3_C_4_H_5_)_2_Mn was as low as 0.185 μmol/min. Therefore, the determination of carrier concentration from the Mn-doped samples was unavailable. This phenomenon could be due to the incorporated Mn atoms in the bandgap of GaN induced states with the deep-level nature to compensate the electrons originating from native donors and thereby reduce the conductivity. Similar results were also reported by Yamamoto *et al*., and they used the high-resistivity Mn-doped layer for high-electron mobility transistors to reduce leakage current^28^. Hwang *et al*. showed that the Fermi level of Mn-doped GaN is shifted downward with Mn doping, which is determined by core-level x-ray photoelectron spectroscopy^29^. In other words, the doped Mn atoms supply holes and compensate the electron carriers. Figure 4 shows the PL spectra obtained from the undoped and Mn-doped GaN layers grown with the different Mn doping concentrations. In Fig. 4(a), the undoped GaN exhibited an intense emission dominated by free excitons or a superposition of the bound and free excitons at approximately 3.43 eV. Two additional peaks at nearly 3.36 and 3.26 eV were observed in the Mn-doped GaN and u-GaN samples. The emission peaks at nearly 3.36 (LO) and 3.26 eV (2LO) were assigned to be the phonon replica of the peak at 3.43 eV, because the Fabry–Pérot interference can be disregarded for the mismatch of the energetic distance between the two peaks^30^. The emission intensity of the peak at 3.43 eV decreased as the Mn-doping level increased, as shown in Fig. 4(b). This phenomenon could be due to the Mn atoms incorporated into GaN formed the Mn-related deep levels and thereby accompany the degradation of crystal quality to induce Shockley–Read–Hall (SRH) recombination (i.e., non-radiative downward transition) events during the light excitation. In addition, a broad band centered at approximately 2.95 eV was also observed in the Mn-doped GaN and u-GaN samples, indicating the transition from shallow donors to totally localized acceptors^31^. The emission band at approximately 1.71 eV is attributed to the second-order peak of the above-mentioned UV emission at 3.43 eV. In addition to the second-order peak, a broad band centered at approximately 1.4 eV was observed from the Mn-doped samples. To eliminate the influence of the second-order peak at 1.71 eV and present better the emission band at approximately 1.4 eV, typical PL spectra were further determined using a long-pass filter with a cutoff wavelength of 560 nm to cut the UV peak. As shown in Fig. 4(b), PL spectra obtained from the Mn-doped GaN samples exhibited a broad emission band centered at approximately 1.4 eV. This emission band could be attributed to the transition of electrons from Mn-related levels within the bandgap to the VB^23,24^. In addition to the evaluation of the PL spectra, electroluminescence (EL) spectra were obtained from the bipolar p-i-n devices, where the Mn-doped GaN layer was sandwiched by the p- and n-GaN layers, to evaluate the optical properties of the Mn-doped GaN. The layer structure of the GaN-based bipolar p-i-n devices was described by the following steps. A 1.25 μm-thick Si-doped n-GaN(n^+^-GaN bottom contact layer) and a 0.6 μm-thick Mn-doped GaN active layer were grown at 1060 and 1000 °C, respectively, on a 3 μm-thick u-GaN/sapphire template. In this epitaxial structure, the (CH_3_C_4_H_5_)_2_Mn with a flow rate of 1.11 μmol/min was used as the Mn-doping source to grow the Mn-doped GaN active layer. The atomic concentration of Mn in the Mn-doped GaN layer was approximately 6 × 10^19^/cm^3^, which was determined via the SIMS from the Evans Analytical Group. Finally, a p-type GaN layer with a thickness of 120 nm and a Si-doped InGaN (n^+^-InGaN topmost tunneling layer) with a thickness of 2 nm were sequentially grown on the Mn-doped GaN layer. The bipolar p-i-n devices with the above-mentioned layer structure were labeled “Device-B” as illustrated in Fig. 5.

**Figure 6:**
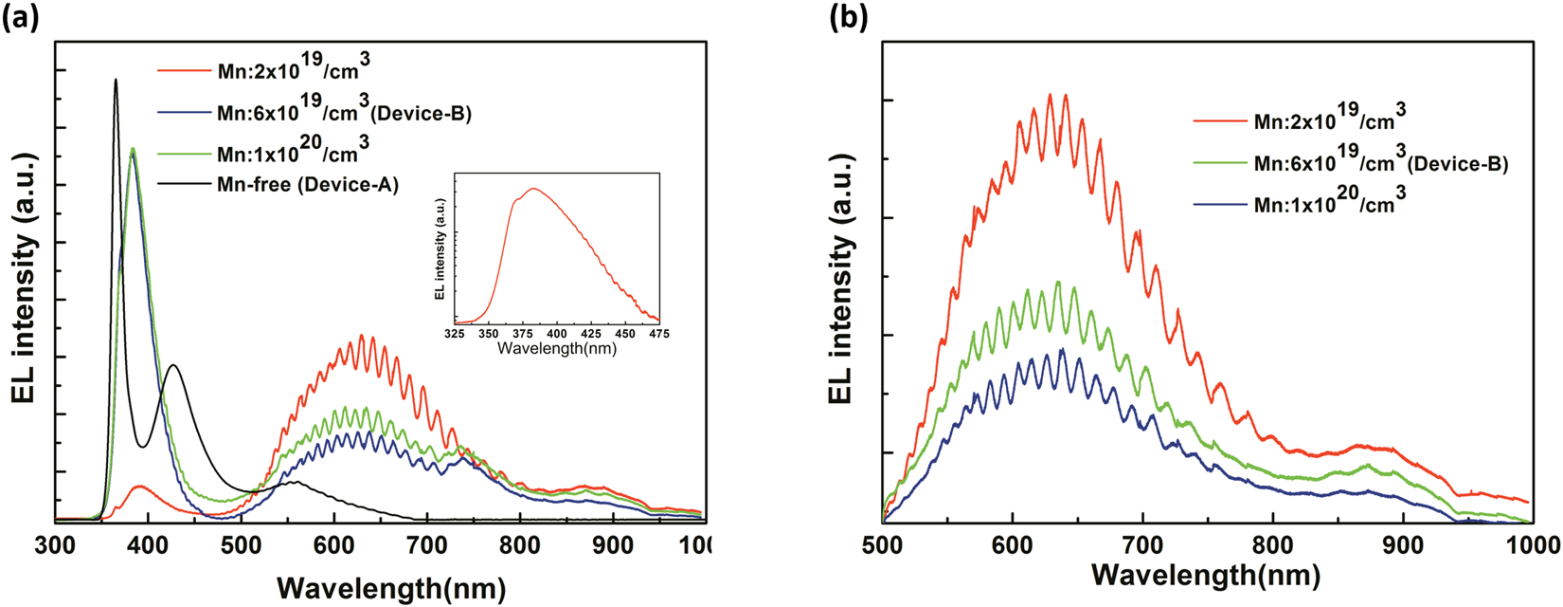
Typical EL spectra obtained at RTs from (**a**) Device-A and Device-B with different Mn doping concentration. (**b**) Typical EL spectra obtained from Device-B after a long-pass filter with a cutoff wavelength of 500 nm. All the sample were operated at a dc current of 100 mA.

For comparison, the Mn-free samples with a 0.6 μm-thick undoped-GaN active layer were also prepared and labeled as “Device-A”. An inductively coupled plasma etching technique was conducted after the epitaxial growth to expose the n^+^-GaN bottom contact layer. An indium tin oxide(ITO) film was deposited in the n^+^-InGaN layer to form the ohmic contact as an anode electrode ohmic contact^32^. Next, a Cr/Au bilayer metal was simultaneously deposited into the exposed n^+^-GaN and the ITO layer to form the cathode and anode electrodes, respectively^33^. The EL spectra were measured using a Synapse charge-coupled device and HORIBA Scientific JY iHR320 imaging spectrometer. Figure 6 depicts the typical EL spectra from Device-A and Device-B with different Mn doping concentration. All the samples were operated under a dc current of 100 mA during the EL measurement. In addition to the samples with doping concentration of 6 × 10^19^/cm^3^ (Device-B), EL spectra taken from samples with doping concentration of 2 × 10^19^/cm^3^ and 1 × 10^20^/cm^3^ were also displayed in Fig. 6. The EL spectra showed that GaN p-i-n structures with different Mn doping concentration in the i-layer (active layer) exhibited similar EL emission only if the doping concentration exceeds 2 × 10^19^/cm^3^. For Device-A, three peaks at 365, 430, and 560 nm were observed as shown in Fig. 6(a). The UV peak at approximately 365 nm is due to the band-edge transition between the CB and VB of GaN. The peak at nearly 430 nm could be attributed to injecting electrons from the Si-doped n-GaN into the Mg-doped p-GaN and hence the recombination with Mg-related states^34,35^. The recombination centers associated with this emission include deep donor states and Mg acceptor levels. A blue band peaked at approximately 430 ± 10 nm obtained from the Mg-doped p-GaN frequently appeared in the RT PL spectra. This emission is attributed to the transition between the defect-related deep donor states and the Mg acceptor levels^34,35^. The yellow luminescence (YL) band peaked at approximately 560 nm, which is a well-known emission of the n-type GaN, was also observed in the EL spectra^36^. The YL band has been previously attributed to a transition involving a complex consisting of a gallium vacancy and a carbon on a nitrogen site^37^. In addition to an UV emission peaked at approximately 380 nm, two broad bands centered at nearly 630 and 740 nm were observed from Device-B. The UV emission (380 nm) band has a shoulder peak at 369 nm as illustrated in the inset of Fig. 6(a). This was attributed to the band-edge transition of GaN. A slightly redshift exhibited by the peak is reasonable compared with Device-A, because the highly Mn-doping concentration could result in a substantial tensile strain in the GaN layer and thereby cause a reduction of bandgap energy. The substitutional Mn atoms with a radius of 0.117 nm incorporated into the lattice to replace the Ga atoms with a radius of 0.126 nm induce a tensile component in the strain field for high Mn-doping concentration. This situation compensates the residual compressive strain because of the lattice mismatch between the sapphire and GaN film. Similar strain effects caused by generating Si-related defects have been reported in the Si-doped GaN layers grown on sapphire substrates^37^. The exact origin of the peak at 380 nm is currently unknown. The transition might involve the Mn-related structural defect states (e.g., interstitial defects or dislocations) within the bandgap of the GaN layer, because the emission peak at 380 nm is lacking in the EL spectrum of Device-A. Particularly, incorporating Mn atoms into the GaN layer may induce extra structural defects to cause the 380 nm emission. The emission band centered at approximately 630 nm was attributed to the transition between the Mn-related IB and CB, and the above-mentioned YL band might be embedded in the emission band (λp ~ 630 nm). The emission band at approximately 740 nm is attributed to the second-order peak of the UV emission (λp ~ 369 nm), and its tail extends between 850 and 950 nm. Typical EL spectra obtained from GaN p-i-n structures with different Mn doping concentration were further performed using a long-pass filter with a cutoff wavelength of 500 nm to cut the UV peak (λp ~ 380 nm) to eliminate the influence of the second-order peak. In Fig. 6(b), two broad emission bands centered at approximately 630 (~1.9 eV) and 880 nm (~1.4 eV) were clearly observed in the EL spectra. The broad emission bands centered at approximately 630 nm could be attributed to the transition of electrons from CB to the partially filled Mn-related IB within the bandgap. Meanwhile, the infrared emission band centered at approximately 880 nm could be attributed to the transition of electrons from the Mn-related IB to the VB. Based on the results, the Mn-related IB band could be suggested, which were first evidenced by electrical pumping to probe the position of the Mn-related energy states, to form at lower than the middle of the GaN bandgap in the Mn-doped GaN. These findings based on the EL spectra were consistent with the previous experimental results based on PL and/or transmittance and theoretical expectation^9,23,38^. In addition, preliminary results showed that the two-photon absorption process via the IB operation could lead to a positive impact on the conversion efficiency of the GaN p-i-n solar cells with the Mn-doped absorption layer^21^.

**Figure 7:**
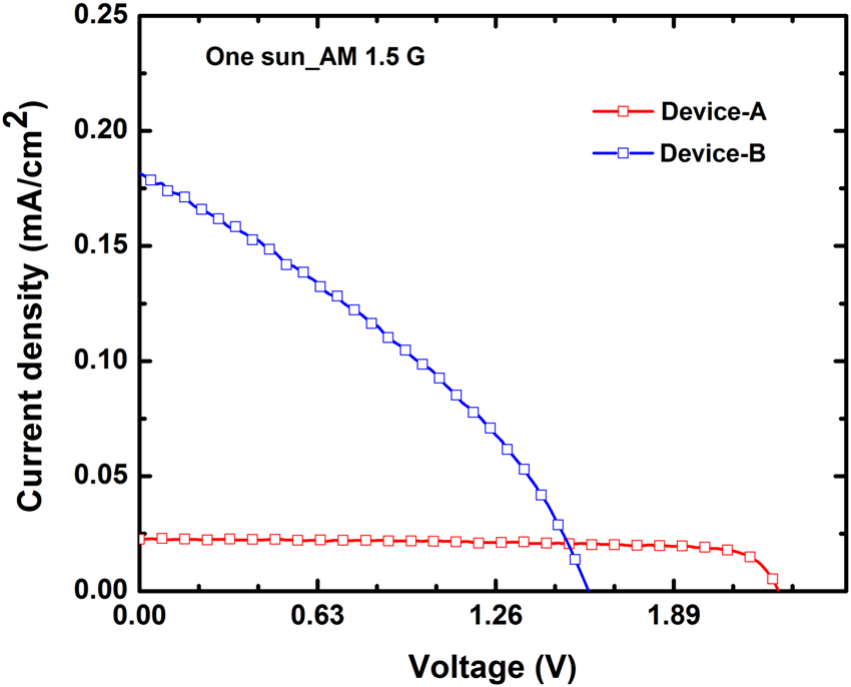
Typical current density–voltage characteristics of Device-A and Device-B under illumination with the AM1.5 G solar condition.

**Figure 8:**
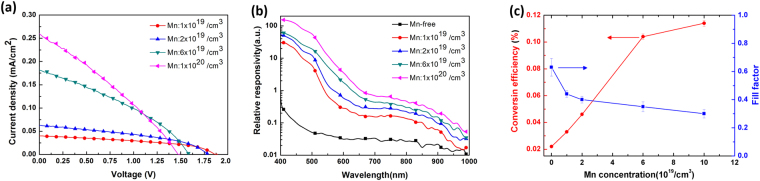
(**a**) Typical current density-voltage characteristics under AM1.5 G illumination. (**b**)Spectral responses in the visible region obtained from GaN IB solar cells with different Mn doping concentrations in the absorption layer. (**c**) Overall conversion efficiency of GaN IB solar cells with different Mn-doping concentrations in the absorption layer.

Figure 7 depicts the typical current density–voltage (J–V) characteristics of Device-A and Device-B under illumination with the AM1.5 G solar condition. A significant photovoltaic response was observed for both devices. A fill factor (FF) of approximately 60% was determined from Device-A considering the J–V characteristics. However, Device-B exhibited a relatively low FF of approximately 45%. The low FF was attributed to the high series resistance of Device-B. This result was consistent with the Hall-effect measurements, where the resistivity of the Mn-doped GaN epitaxial layers was larger than that of the u-GaN epitaxial layers. In terms of the short-circuit current density (*J*_sc_) and open-circuit voltage (*V*_oc_), Device-B exhibited a higher *J*_sc_ and a lower *V*_oc_ compared with Device-A. The increased *J*_sc_ can be attributed to the additional absorption of photons with energies below the GaN bandgap. The measured typical *V*_oc_ were 2.26 and 1.58 eV for Device-A and Device-B, respectively. The reduction of V_oc_ could be due to the incorporation of Mn impurities causes the material quality degradation and then the electricity leakage. The Mn-related states within the bandgap may form IB-associated structural defect states. In principle, the IB has a quasi-Fermi level within the bandgap. In terms of external electrodes (p- or n-type), the *V*_oc_ of a solar cell with an IB absorption layer is equal to a solar cell without an IB absorption layer because *V*_oc_ is theoretically obtained based on a split between the quasi-Fermi levels of the electron and hole. However, the *V*_oc_ for a practical solar cell with an IB absorption layer is frequently lower than that of the IB-free solar cell because of the undesirable connections between the defect-related trap state and the quasi-Fermi level in the IB^10–14^. Particularly, the structural defect-related trap states within the bandgap might result in a poor isolation of the quasi-Fermi level of IB from the quasi-Fermi levels of the free electron and hole. Therefore, the high Mn concentrations in the GaN solar cells could degrade the *V*_oc_ because of the defect states within the bandgap-behaving leakage source. The increased photocurrent in Device-B was a key point in improving the conversion efficiency via the IB operation, although high Mn doping may lead to the reduction of the *V*_oc_ of solar cells. Devices with different Mn doping levels in the absorption layer were also prepared to clarify the effect of Mn-doping concentration on the conversation efficiency. The layer structures in these devices were the same as in the Device-B. The typical current density-voltage characteristics of Mn-doped GaN IB solar cells under one-sun AM 1.5 G illumination are shown in Fig. 8(a). As one can see that J_sc_ increase with the concentrations of Mn doping in the absorption layer. However, when the Mn concentration increased, the V_oc_ decreased. The increase in J_sc_ was enhanced by the absorption of sub-bandgap photons because the density of Mn-related IB states within the band gap of GaN increased with the Mn doping concentration in the absorption layer. This contention could be evidenced by the visible spectral responses obtained from devices with different Mn doping concentrations in the absorption layer, as shown in Fig. 8(b). Since the negative effect of Mn doping on the material quality remains in the experimental devices, the V_oc_ and FF exhibited an opposite trend compared with the J_sc_. However, dramatic increase in the photocurrent of cells without considerable voltage reduction resulted in a marked enhancement in the conversion efficiency, as shown in Fig. 8(c). These results suggested that the Mn-related states within the bandgap of GaN could be potentially employed to realize IBSCs with high efficiency.

## Conclusions

In summary, optical characterization based on the EL, PL, and transmittance performed on the Mn-doped GaN layers indicated that considerable Mn-related energy states existed in the bandgap and exhibited a significant effect on optical transition. The position of the Mn-related energy states was first evaluated via the EL spectra obtained from the bipolar devices with the Mn-doped GaN as an active layer under electrical pumping. This below bandgap transition also suggested that the Mn-related states within the bandgap of GaN form an IB to realize an IBSCs with improved conversion efficiency.

## Electronic supplementary material

Supplementary Information
