# Supplementary material for: GaN intermediate band solar cells with Mn-doped absorption layer
Source: Sci Rep. 2018 Jun 5;8:8641. doi: 10.1038/s41598-018-27005-z (PMC5988796; doi:10.1038/s41598-018-27005-z)
Supplement: Supplementary file 1 — Supplementary Information [file 41598_2018_27005_MOESM1_ESM.docx]

GaN intermediate band solar cells with Mn-doped absorption layer

**Ming-Lun Lee^1^, Feng-Wen Huang^2^, Po-Cheng Chen^2^, and Jinn-Kong Sheu^2,*^**

^1^Department of Electro-Optical Engineering, Southern Taiwan University of Science and Technology, Tainan City 71001, Taiwan

^2^Department of Photonics and Advanced Optoelectronic Technology Center, National Cheng Kung University, Tainan City 70101, Taiwan.

*Contact Email: [jksheu@mail.ncku.edu.tw](mailto:jksheu@mail.ncku.edu.tw) (J. K. Sheu)

**Supporting information:**

Figure S1 displays the Mn ion counts of the Mn-doped GaN determined by SIMS as a function of the growth temperature. Other epitaxial growth parameters, except for the growth temperature, were fixed. In this case, the growth pressure and the (CH_3_C_4_H_5_)_2_Mn molar flow rate were 100 torr and 1.11 µmol/min, respectively. The relative Mn atom concentration was nearly maintained at a constantas the growth temperatures ranged from 950 °C to 1010 °C. This result implies that the incorporation efficiency of Mn atoms in GaN is dominated by the mass transfer mechanism in this temperature range. However, the relative concentration of Mn atoms in GaN decreases sharply as the growth temperature increased to 1040 °C. The significant reduction of the incorporation efficiency of Mn in GaN at a relatively high growth temperature could be attributed to the reevaporation effect of Mn atoms from the growth surface. A similar effect was observed when the p-type GaAs-based materials were grown by MOVPE using dimethylzinc or diethylzinc as a precursor. The material quality of the GaN epitaxial layer would degrade if the growth temperature is low, although the incorporation efficiency of Mn atoms in GaN can be intuitively increased by decreasing the growth temperature. Therefore, the growth temperature of the Mn-doped GaN epitaxial layers was fixed at 1010 °C for the subsequent experiments. In addition to the abovementioned growth parameters, the effect of growth pressure on incorporating Mn in GaN was also investigated.


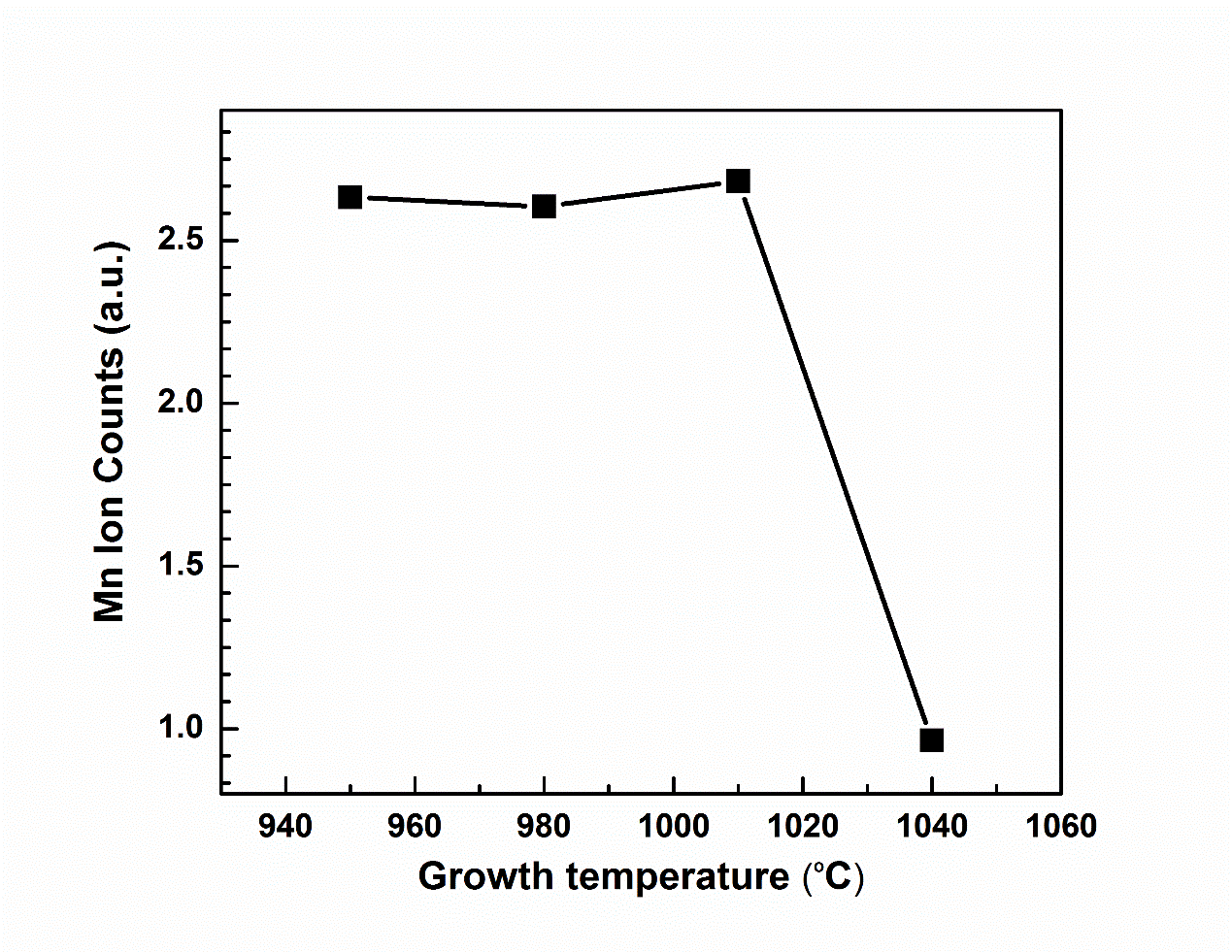


Figure S1. Mn ion counts of the Mn-doped GaN determined by SIMS as a function of the growth temperature.

Figure S2 depicts the Mn ion counts of Mn-doped GaN determined by SIMS as a function of the growth pressure. In this case, the growth temperature and (CH_3_C_4_H_5_)_2_Mn flow rate were fixed at 1010 °C and 1.11 µmol/min, respectively. In Figure S2, the relative counts of Mn atoms in GaN decrease with an increase of the growth pressure. The result could be attributed to the pre-reaction of (CH_3_C_4_H_5_)_2_Mn with NH_3_ before they reach the growth surface. The probability of collision (or interaction) between the gas-phase precursors decreases and thereby suppresses the pre-reaction as the growth pressure decreases. Therefore, the incorporation efficiency of Mn atoms in the GaN epitaxial layer could be increased.


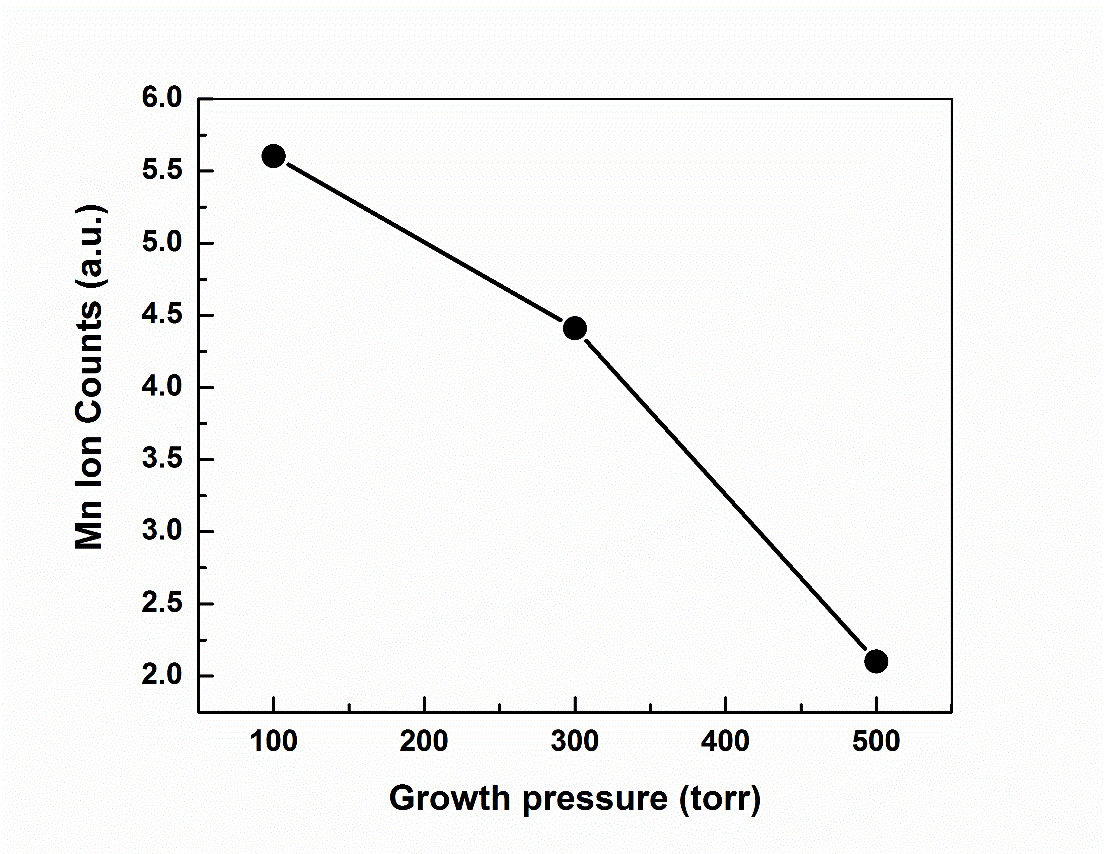


Figure S2. Mn ion counts of the Mn-doped GaN determined by SIMS as a function of the growth pressure.

Figure S3 illustrates the typical photos of the GaN epitaxial layers doped with (CH_3_C_4_H_5_)_2_Mn molar flow rates of 0, 0.185, and 1.85 µmol/min from left to right. The color appearance changed from transparent to orange when the Mn dopant concentration increased gradually. These colors could be due to the absorptions are caused by the d–d transitions in the substitutional divalent transition metal atoms, indicating that the Mn-related energy states in the bandgap of GaN absorb visible light with a wavelength less than 650 nm. This contention can be indirectly clarified by the transmission spectra obtained from the Mn-doped GaN samples.


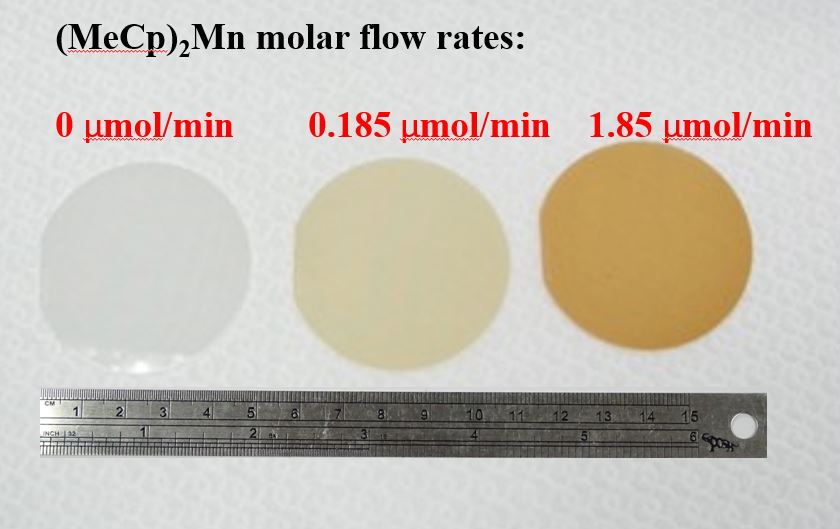


Figure S3. Typical photos of the GaN epitaxial layers doped with the (CH_3_C_4_H_5_)_2_Mn molar flow rates of 0, 0.185, and 1.85 µmol/min from left to right.


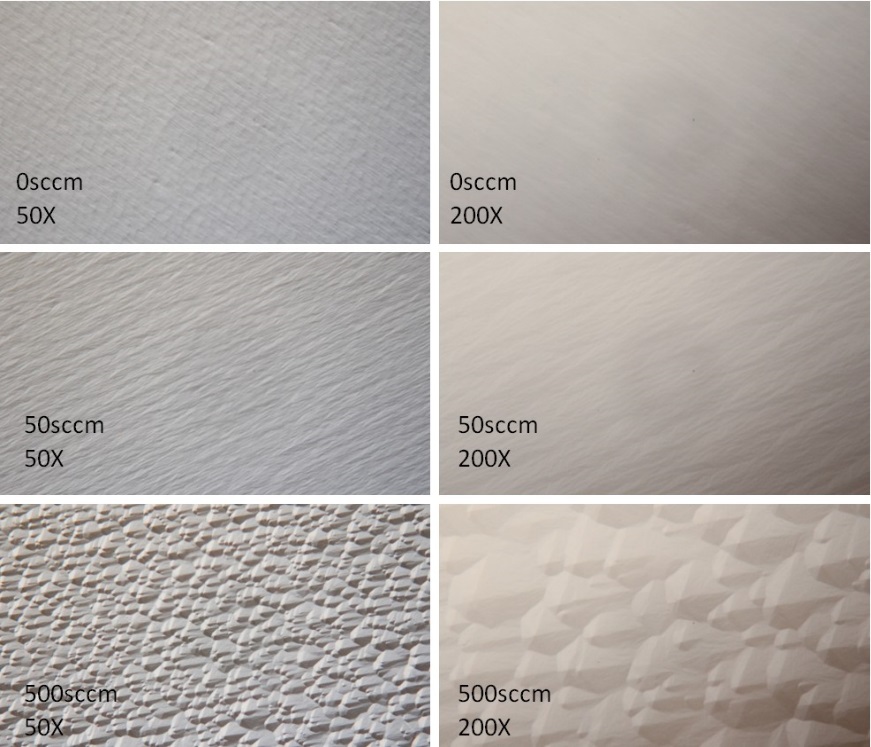


Figure S4. Typical photos of the Mn-doped GaN grown with different molar flow rates of (CH_3_C_4_H_5_)_2_Mn captured by the optical microscope at 50× and 200× magnification. The 0 sccm means u-GaN. The 50 and 500 sccm denote the Mn-doped GaN layers grown with the (CH_3_C_4_H_5_)_2_Mn molar flow rates of 0.185 and 1.85 μmol/min, respectively.

Figure S4 presents the typical photos obtained from the Mn-doped GaN grown with different (CH_3_C_4_H_5_)_2_Mn molar flow rates (0, 0.185, and 1.85 µmol/min) under the optical microscopy at 50× and 200× magnification. The u-GaN and Mn-doped GaN epitaxial layers were specular to the naked eye. The Mn-doped epitaxial layers were orange in color when the (CH_3_C_4_H_5_)_2_Mn molar flow rate was increased to 1.85 µmol/min, and their surface roughness increased because of the hillock with a hexagonal cone shape. The rough surface could be due to the increase in crystal stress, and then the growth mode would become a Stranski–Krastanov and/or Volmer–Weber growth mode because of the significant difference of radius between the GaN and Mn atoms as incorporating Mn atoms was increased by increasing the (CH_3_C_4_H_5_)_2_Mn flow rate.

Figure S5 depicts the typical atomic force microscopy (AFM) images of the Mn-doped GaN grown with different (CH_3_C_4_H_5_)_2_Mn molar flow rates (0, 0.185, and 1.85 μmol/min). The u-GaN and lightly Mn-doped (0.185 μmol/min) GaN films exhibited a clear step flow pattern, but the heavily doped (1.85 μmol/min) GaN exhibited an extremely rough morphology. The root mean square (RMS) roughness was 0.6, 0.9, and 1.4 nm for the undoped, lightly, and heavily Mn-doped GaN films, respectively.


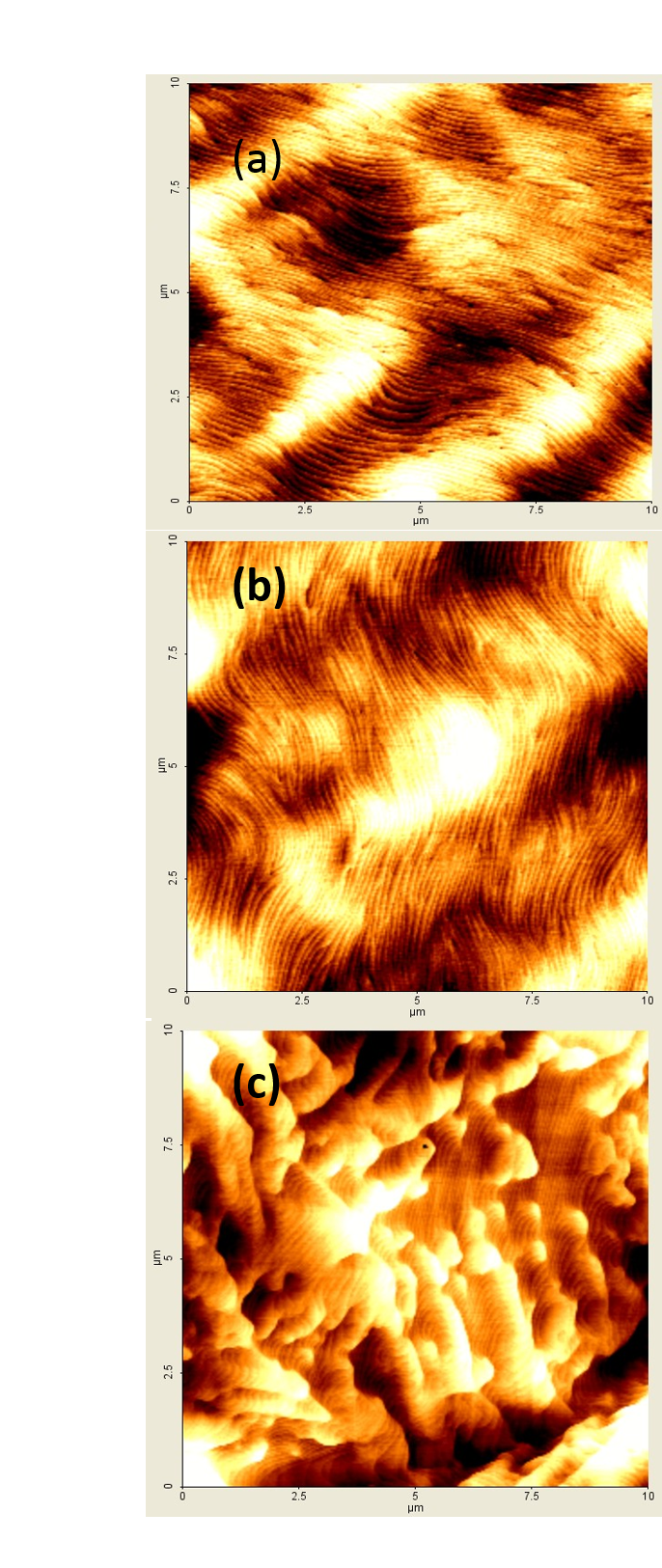


Figure S5. Typical AFM images of the Mn-doped GaN grown with different (CH_3_C_4_H_5_)_2_Mn molar flow rates: (a) 0, (b) 0.185, and (c) 1.85 μmol/min.


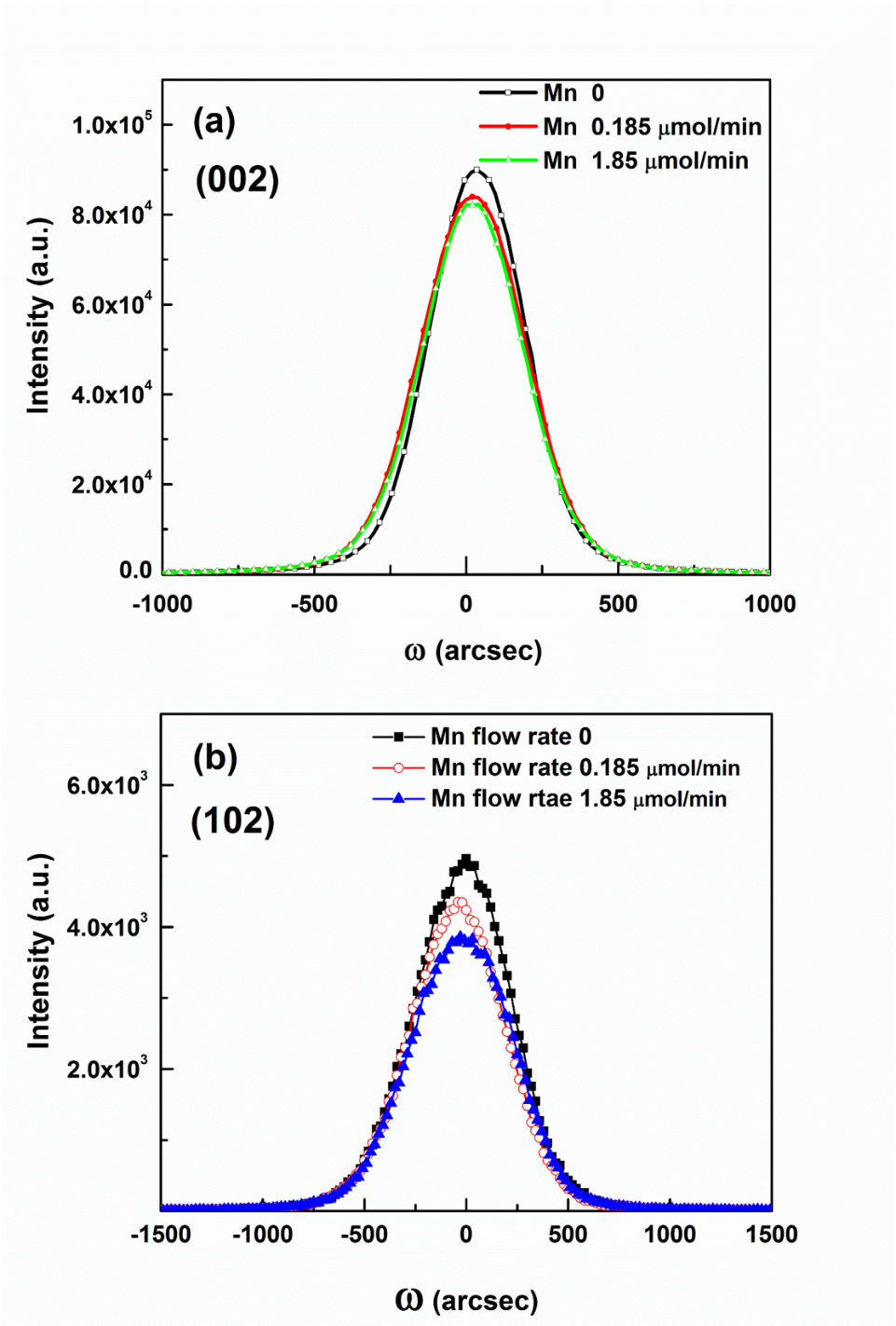


Figure S6. (a) (002) and (b) (102) XRC spectra for the GaN epitaxial layers at different Mn-doping levels.

The result indicated that the morphology changed significantly by increasing the incorporation of Mn, leading to transforming the growth mode. The crystal quality of the Mn-doped GaN films was also characterized using the double-crystal X-ray rocking curve (XRC) analysis.

Figures S6 (a) and (b) illustrate the (002) and (102) XRC spectra, respectively, for the GaN epitaxial layers with different Mn-doping levels. A slight difference in spectrum width could be observed between the u-GaN and Mn-doped GaN films. The full width at half maximum (FWHM) of the XRC spectra at (002) plane obtained from the GaN epitaxial layers grown with the (CH_3_C_4_H_5_)_2_Mn molar flow rates of 0, 0.185, and 1.85 μmol/min were approximately 375, 410, and 404 arcsec, respectively. The FWHM of the XRC spectra at (102) plane for GaN films grown with the (CH_3_C_4_H_5_)_2_Mn flow rates of 0, 0.185, and 1.85 μmol/min were 545, 550, and 600 arcsec, correspondingly. The marked difference in the electrical and optical properties was observed between the undoped and Mn-doped GaN, although the Mn dopants in GaN did not result in significantly different XRC spectra compared with the undoped samples. In this study, the u-GaN epitaxial layers grown on sapphire substrate exhibited an n-type conduction, and the typical electron concentration determined by Hall-effect measurement was approximately 5×10^16^ cm^−3^. The Hall effect measurements indicated that the resistivity of the Mn-doped GaN layers was extremely high exceeding 10^11^ Ω/square, which is the measurement limit of our setup, even when the doping flow rates of (CH_3_C_4_H_5_)_2_Mn was as low as 0.185 μmol/min. Therefore, the determination of carrier concentration from the Mn-doped samples was unavailable. This phenomenon could be due to the incorporated Mn atoms in the bandgap of GaN induced defect states with the deep-level nature to compensate the electrons originating from native donors and thereby reduce the conductivity.
